# Society Meetings

**Published:** 1903-06

**Authors:** 


### SOCIETY MEETINGS.

The National Confederation of State Medical Examining and Licensing Boards held one of the most important meetings in its history at New Orleans, May 4, 1903. A special feature of the preliminary exercises was the address of Dr. Rudolph Matas, of Tulane University, who not only welcomed the visitors in appropriate terms, but presented a comprehensive review of the whole field of state licensure, that cannot fail to benefit the cause of higher medical education and advance the interests of state control. Other papers and addresses of great interest were likewise presented, and when the proceedings are published attention will be attracted to important features developing along the lines of state examinations for license to practise. Dr. Henry Beates, Jr., of Philadelphia, was elected president and Dr. F. A. Larue, of New Orleans, was chosen secretary, Dr. Suiter having declined to serve further in the latter capacity.

---

The Medical Society of the County of Erie will hold its regular semiannual meeting on Tuesday, June 11, 1903, beginning at 10 o'clock a.m. The place of meeting is in the parlors of the Y.M.C.A., corner of Pearl and Mohawk Streets, Buffalo. The president, Dr. Ernest Wende, and the secretary, Dr. Franklin C. Gram, are preparing a program that should prove of interest to physicians, lawyers, clergymen and pharmacists, all of whom are invited to attend.

---

The sixth triennial congress of American Physicians and Surgeons was held at Washington, D.C., May 12-14, 1903. It is composed of sixteen constituent special societies, among which is the American Neurological Society, which was presided over by Dr. James Wright Putnam, of Buffalo. All of the societies attended to their own programs in the morning of each day and in the afternoon the general sessions of the congress were held. A number of distinguished foreigners were present by invitation, among whom were Prof. J. von Mikulicz-Radecki, of Breslau, Prof. C. A. Ewald, of Berlin, Prof. Hans Kehr, of Holberstadt, Prof. Hermann Tillmans, of Leipsic, and Mr. B. S. A. Moynihan, of Leeds, England. It is a matter of common remark that the sixth congress was quite the most successful gathering in the history of the organisation.

---

THE Buffalo Academy of Medicine held meetings during the months of April and May, 1903, as follows:

*Section on Medicine.*—Tuesday evening, April 28. Program: Report of twenty cases of pernicious anemia, Charles G. Stockton and Albert E. Woehnert.

Wednesday evening, May 13. Program: The medical and surgical treatment of Bright's disease, Charles S. Jewett.

*Section on Surgery.*—Saturday evening, May 2. Program: Surgical experiences in the Philippines, Maj. Charles Lynch, Surgeon U. S. Army.

*Section on Pathology.*—Tuesday evening, May 19. Program: Infectious endocarditis, with report of cases, Albert E. Woehnert.

*Section on Obstetrics and Gynecology.*—Tuesday evening, May 26. Program: Should the uterus be removed when operating for pus tubes? Matthew D. Mann; Prophylaxis in obstetrics, Ludwig Schroeter.

